# A comprehensive dataset of U.S. federal laws (1789–2022)

**DOI:** 10.1038/s41597-023-02758-z

**Published:** 2024-01-02

**Authors:** Brian Libgober

**Affiliations:** 1 Northwestern University, Department of Political Science and School of Law, Evanston, IL 60202 USA; 2 Yale Law School, Yale University, New Haven, CT 06511 USA

**Keywords:** Politics, Law, Government

## Abstract

U.S. federal laws figure importantly in many research projects in political science, law, sociology, economics, and other disciplines. Despite their prominence, there is no authoritative, current, and comprehensive dataset of U.S. federal laws. In part, this is because such laws have been enacted over hundreds of years, resulting in a complicated patchwork of documents published in numerous and inconsistent formats. As a simplification, many scholars have relied upon selective lists of major legislative enactments or complete lists of relatively recent enactments. Here, I report on an effort to transparently and reproducibly assemble a complete database of US laws and their revision histories by combining data from HeinOnline, the Governmental Printing Office, and the National Archives and Records Administration. The result is a database of 49,746 laws spanning 1789 to 2022.

## Background & Summary

What is a law? The Oxford English Dictionary defines it as “a rule or system of rules recognized by a country or community as regulating the actions of its members and enforced by the imposition of penalties.”^1^ So defined, laws in the United States can originate from many sources, including courts, the President, executive agencies, and so forth, both at the state and the national-level. Yet when scholars speak more formally about the set of “U.S. laws,” they often intend to refer to a smaller set of authoritative documents. In particular, they frequently mean those federal laws enacted by the two Congressional chambers, potentially over the President’s veto, through the legislative process outlined in Article 1 Section 7 of the U.S. Constitution. When Congress acts legislatively in this fashion, it may do so by issuing a variety of documents that go by different names, have different formal features, and follow different internal processes. These sub-genre distinctions can and indeed have changed over time. At present writing, the set of allowable legislation include bills, joint resolutions, concurrent resolutions, and simple resolutions. Of these, only bills and joint resolutions have the general and binding character that one typically associates with laws. And even then, there are exceptions and sub-divisions within these categories. Last year Congress enacted a private law allowing Alaska-woman Rebecca Trimble to obtain permanent resident status, despite the fact the paperwork her adoptive parents had used to bring her to the United States from Mexico had been defective. Such “private bills” granting relief in one-off cases are typically not what scholars have in mind when one refers to “laws.” Nor are joint resolutions approving constitutional amendments which are not ratified by the states really laws (e.g. the Equal Rights Amendment). Despite this esoteric mess of terminology, the set of enacted bills and joint resolutions is a relatively precise definition of what scholars *should* mean when they talk about the set of U.S. federal laws, that is to say federal legislative enactments with a binding public character. Surprisingly, there is not, at least to the authors knowledge, a transparent, fully comprehensive, and almost current database of these documents.

Given the importance of U.S. federal lawmaking, it is surprising that no data source meets these goals of coverage and transparency. Yet it is worth detailing the numerous and painstaking data collection efforts that have come increasingly close. My focus is on public, scholarly data collection efforts that produce the kinds of tables that are appropriate for statistical analysis, rather than proprietary databases such as ProQuest Congressional or HeinOnline which are designed with such access constraints that researchers cannot easily use their contents for many important research purposes or validate what these datasets deliver. Some of the earliest existing data responsive to these criteria comes from a key debate in American political science about the consequences of divided government. In particular, scholars have wondered whether the state of unified government, where the President and Congress are controlled by the same party, is better or worse for legislative productivity than divided government, where these organs are not exclusively in controlled by the same political party. This debate was initiated by Mayhew (1991), who engaged this question quantitatively through a dataset of “major” legislative enactments. With slight simplification, Mayhew identified those pieces of legislation that were substantial through analyzing newspaper articles that summarized the achievements of the past legislative session, as we well as an examination of specialized historical works that retrospectively evaluated the importance of key pieces of legislation in a large number of policy areas. Mayhew’s list of major enactments has been updated annually since its original publication in 1991 and is, at present writing, current from 1947 through 2022. Of course, this coverage of major legislative enactments is obviously limited as a database of all federal laws, both because it does not cover laws that fail to draw the attention of editorial pages, nor does it cover laws enacted during the New Deal or earlier. Three issues implicit in Mayhew’s pathbreaking data collection and analysis have continued to bedevil social scientists working in this area: transparency and reproducibility of inclusion or exclusion criteria, limited temporal coverage, and comprehensiveness within covered time-periods^2^.

There have been several follow-up efforts to Mayhew’s project that are notable for their attempt to extend data on legislative enactments in various ways. Howell *et al*.^3^ collect data on 17,633 public laws enacted between 1945 and 1994 and categorize them into four bins of importance, finding critical differences in terms of legislative productivity across unified and divided government according to legislative significance^3^. Clinton and Lapinski^4^ enter the same debate by introducing an IRT model for estimating the importance of enacted laws between 1877 and 1994^4^, and have subsequently used these same data and estimates to reconsider questions about the interpretation of roll-call votes as a measure of political ideology^5–7^. The Clinton-Lapinski model of legislative importance leverages an even more comprehensive assembly than the Howell *et al*. effort, comprising some 37,766 law going all the way back to the post-Civil War era. Yet to the authors knowledge, this comprehensive dataset has not been updated to include laws enacted in the last two decades. Nor does it extend backwards into the first century of the U.S. Republic. As we shall see, that leaves about 25% of all laws and almost 100 years of law-making uncovered.

Several other major research projects partially address the issue of currency and comprehensiveness, but neither in a completely satisfactory way. It is also worth emphasizing that these projects all use somewhat different approaches to identifying laws, which are mostly but not perfectly inter-operable. The Policy Agendas Project, for example, is a long-standing collaborative research effort aimed at building multiple datasets for tracking and comparing which issues manage to attract the attention of government across. It covers many national and subnational systems, including the United States federal government. In particular, their database of bills covers “more than 400,000 bills introduced by the U.S. Congress.” Importantly, they also have coded each bill according to the subject-matter coding system of the larger Policy Agenda Project. Their focus on covering the subject matter of lawmaking is an important undertaking, although arduous, and it is unsurprising given the difficulties of this undertaking that they have only managed to cover the period 1947–2022.

Another project with relevant and similar prior data collection is due to Ansolabehere, Palmer, and Schneer^8,9^. Their data collection, which was conducted under the auspices of an undergraduate course at Harvard called “What Has Congress Done,” crowd-sourced the task of identifying significant legislation to students, each of whom was responsible for producing a list of significant legislation enacted under each of 22 potentially assigned decades. The researchers provided a list of secondary sources, as well as guidance and quality control efforts. Similar to Mayhew, their criteria for inclusion were (a) was the law considered significant at the time of enactment, and (b) does the law seem significant in historical perspective. Because of their differing methods, Mayhew and Ansolabhere, Palmer and Schneer do not completely overlap in the 1947–2022 period as databases of significant legislation. Although the temporal coverage for the Ansolabehere, Palmer, and Schneer dataset is very long, it contains a small percentage of all laws enacted by the U.S. Congress. None of the so-far mentioned sources, therefore, comes anywhere near a comprehensive dataset of U.S. laws.

This project seeks to build a comprehensive and easily updated list of U.S. laws and their revision histories based on publicly available sources. The primary source we leverage is the *Statutes at Large*, which has been published continuously since 1845, and whose early issues contain the earliest legislative enactments. The most recent issue of the *Statutes at Large* is from 2017, and it is published with about a six year delay. Even though the *Statutes at Large* are not physically or electronically available for the last six years, the National Archives and Records Administration provides citations to page numbers almost immediately. For example, on September 25, 2023 it was possible to get the statute at large citation of a law enacted only three days prior (https://web.archive.org/web/20230925173228/https://www.archives.83 gov/federal-register/laws/current.html).

Although the *Statutes at Large* are well-known to scholars, this source have not until recently been “born digital.” As a result, when electronic data on these laws are available, they have typical issues of digitized text data, despite the very high importance of these documents. Indeed, it is somewhat surprising that the U.S. government does not make electronic copies of the *Statutes at Large* available prior to 1951, despite the fact that there were 64 earlier volumes containing laws that in many cases have not been amended. In legal practice, not having these documents digitized usually works out well enough, because of ongoing public and private efforts to develop codices of laws, however there are occasional issues of interpretation where returning to the actual text of the law in the *Statutes at Large* is necessary. In addressing such esoteric issues, legal scholars and lawyers often leverage use an electronic database called HeinOnline, which has digitized all these paper volumes in their entirety. Fortunately, they also provide meta data on the tables of contents of these volumes. For years in which the government-provides meta-data about laws through its Governmental Printing Office, we can rely on this meta-data to construct a list of laws. For older years that the GPO has not yet reached, we rely on the meta-data provided by HeinOnline. For more recent years where the *Statutes at Large* do not yet exist, we rely on data on the National Archives website to supplement and bring our dataset to currency (https://www.archives.gov/federal-register/laws). Per that site, “After the President signs a bill into law, it is delivered to the Office of the Federal Register (OFR)[, a division of the National Archives], where editors: assign a Public Law Number[,] prepare it for publication as a Slip Law[,] include it in the next edition of the United States *Statutes at Large*.”

Our dataset covers laws enacted from 1789 to 2022, the year prior to publication, and contains 49,746 entries. A Github repository provides linked to this publication provides code to update the set of laws to the present.

## Methods

We rely on three primary sources for our comprehensive database of U.S. laws: the oldest meta data for the U.S. *Statutes at Large* disseminated via HeinOnline, similar and more recent meta data through the Governmental Printing Office, and finally the last six years of law-making as described by the National Archives’ website.

We began our data collection by scraping the table of contents of each volume of the *Statutes at Large*, excepting volumes 6-8 which contain a collection of early treaties and private laws. Importantly, examination of these tables of contents quickly reveal weaknesses in many approaches to uniquely identifying laws. In particular, the public law numbers used today were not used consistently prior to the 20th century. There were also periods in the 20th century where public law numbers were repeated across sessions of the same convening of Congress, as opposed to more recent practice where the new session of a particular Congress starts counting where it left off in the last session. Citing pages and volumes of the *Statutes at Large*, probably the most common practice in ordinary government and legal use, does not effectively disambiguate laws because it is not uncommon for two or even three laws to share the same starting page. Presumably, individuals who care about the text of a particular law know how to fill in the gaps caused by this ambiguity, however for the purposes of building *databases* this is clearly not ideal. Disambiguating based on the names of the laws is possible, but potentially mistake prone as different sources may use different capitalizations, punctuation, and spacing, short versus long names, and other issues typical of text data.

These caveats aside, examination of the published pages of the *Statutes at Large* as well as the table of contents reveals that laws *are* identified using an (almost perfectly) precise numerical citation systems, however the exact system that works depends on the era. In particular,

- The currently practiced identification system (“Late regime”) begins on Jan 7, 1959 with the 86th Congress. In this system, laws are identified by the number of the Congress and the law number within Congress. An example is “Public Law 105-89, An Act: to promote the adoption of children in foster care.” There is only one Public Law 105-89, it uniquely refers to that titled law, and the *Statutes at Large* has published the text of the law with that identifier.
- Initially, laws were identified by a different system (“Early Regime”). From the 1st through the 56th Congress, which concluded March 3, 1901, laws were identified in the *Statutes at Large* by chapter as well as Congress and session (e.g. “Chapter 15, 56 Congress, Session 1, An Act: Relating to Cuban vessels.”). All laws in the *Statutes at Large* are published with this information during the early period. The Early Regime and Later Regime are not interoperable, as the published pages of the *Statutes at Large* in the early period do not provide public law numbers and the published pages in later period do not provide chapters. The early and later periods use fundamentally different citation systems.
- For the 57th through 85th congress, the *Statutes at Large* uses both systems, but not always consistently, and not consistently in the same way as they would before or after. Indeed, a key difficulty in this period is that chapters and public laws are sometimes recycled within one Congress over multiple sessions of a particular Congress. And worse, in just a few instances, the same session. Indeed, PL 65-246 refers to both a law “Providing for the transportation from the District of Columbia of governmental employees whose services no longer are required.” and also a law intended “To authorize the sale of certain lands to school district numbered twenty-eight, of Missoula County, Montana.” The law identification system that superficially seems to be inter-operable with both the prior and subsequent systems does not really work very well with either. Careful attention to chapters, public laws, and sessions does disambiguate nearly all laws, however these details are very particular and subtle. Caution is needed.

Following these citation practices, we used a rule-based parser to extract key pieces of information from scraped meta-data and build a database of laws. Technical validation efforts revealed that there were occasional errors of transcription in our source data, for example mis-identification of chapters associated with laws or including page numbers that were not correct. Those errors that were found in the source data we corrected to conform with the text image captured on the HeinOnline site, although our guess is that there are others which have not been caught. Such issues highlight the needs for *transparency* and *reproducibility* in the process of data collection and data correction, as it is unlikely that any effort that lack either will avoid such issues.

## Data Records

The primary dataset^10^ we provide is publicly available at https://osf.io/qa289. Additional data of interest used for validation is available within the files repository, available at https://osf.io/mrghc.

## Technical Validation

We offer two primary approaches to support the technical validation of our dataset^10^. The first is to compare the total counts of laws by Congress against total counts previously published by social scientists. The second validation exercise involves showing that we cover well a particularly comprehensive dataset of laws published by the Office of Revision Counsel, the agency within Congress responsible for preparing the U.S. Code.

**Fig. 1:**
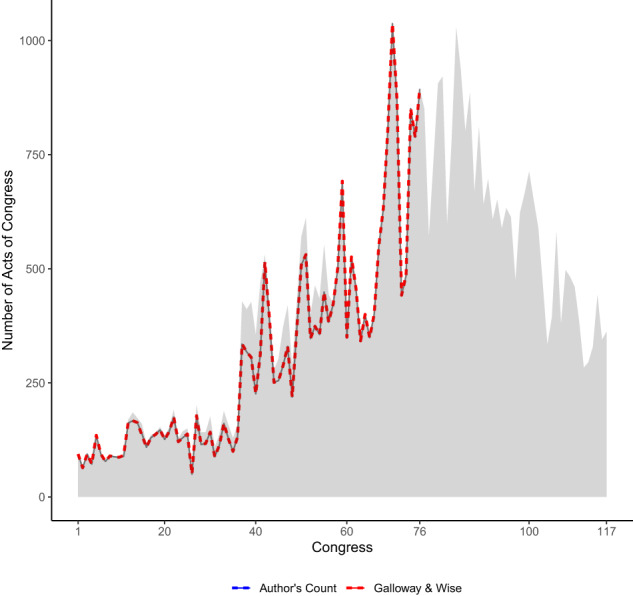
Number of Acts of Congress by Meeting of Congress. Shading reflects total counts of all laws in our dataset, while the colored lines show the overlap in directly comparable counts between subsets of Galloway and Wise and our data.

The reference counts for total laws that we rely on comes from Appendix F reported in Galloway and Wise’s History of the House of Representatives. It was the same set of reference counts previously used for validation and analysis by Ansolabhere, Palmer, and Schneer^8^. Our definition of law is slightly more capacious than Galloway and Wise’s, in the sense that we also consider “Joint Resolutions” laws, and the authors admit their own counts combine acts and resolutions (the latter of which are not, in our view, best understood as laws) from the 77th Congress and after. Nevertheless, it is possible to subset our data in such a way as to include the same set of documents as Galloway and Wise cover, and thereby constitute an appropriate test of technical validity. Figure 1 compares the counts for this key subset of our data. Note that in total Galloway and Wise count 23,155 laws between the 1st and 76th Congresses, while we would peg the figure at 23,152, or three laws fewer (≈0.13% of the total). The true discrepancy between our counts and Appendix F is somewhat greater, because in some years our counts are higher than Galloway & Wise’s and in some years lower. However, in all but six Congresses, our counts are identical. Summing up the absolute value of the difference in counts in these six Congresses, we arrive at a total difference in counts of 11, again a very small difference on a percentage basis. An undergraduate research assistant further investigated the difference in counts of laws in the 11th, 15th, and 34th Congress. In each case, the number of laws that the research assistant counted in that Congress was the same as the number we provided, and we were unable to determine why there was a discrepancy between the Galloway and Wise total and our own.

Our second validation exercise works by attempting to match our database of laws against a particularly large collection of U.S. laws. The dataset we use is the table of official popular names for Congressional enactments as produced by the Office of Revision Counsel (https://uscode.house.gov/popularnames/popularnames.htm). This table catalogues in its rows a set of 13,170 popular or short-names for official legal acts. The table’s rows have entries such as “Obamacare,” which directs readers to see another row on the “Patient Protection and Affordable Care Act.” In the row corresponding to the “Patient Protection and Affordable Care Act,” the table provide a citation “Pub. L. 111-148, Mar. 23, 2010,124 Stat. 119.” Although the table contains many hundreds of rows cross-referencing entries, which are not directly useful for testing, the source nevertheless can be used to construct a very strong validation test of our source data. In particular, it has very long temporal coverage, especially as compared with most datasets of important legislation, and it has very deep coverage, as it cites many thousands more laws than most other datasets. The main alternative contender with equivalent temporal coverage, the crowd-sourced dataset produced by Ansolabhere, Palmer, and Schneer^8^, has many thousands less laws and follows considerably less consistent citation practices than the Office of Revision Counsel.

That said, while the Popular Names Table is a very good source for validating, there are a few difficulties. First, and perhaps most importantly, the popular names table includes names for specific *pieces* of legislation that are part of larger legislative enactments. For example, the Biggert-Waters Flood Insurance Reform Act of 2012 refers just to subtitle A of Title II of Division F of a 584-page highway bill enacted in the same year (Public Law 112-141). While our database of laws does include 112-141 as a law, it does not include titles and sub-titles like the Biggert-Waters Act, so such rows need to be excluded from our matching exercise. Secondly, even after screening out these subpart the popular names table also includes a number of suspicious entries. These involve issues such as two names referring to the same public law number but claiming different enactment dates. This issue impacts some 745 entries, which is a considerable number, and there are also other similarly suspicious issues involving citations. Because of these and several other issues, and also because the number of laws with popular names is considerably smaller than the number of laws, we view the key test of our dataset as what proportion of the *unproblematic entries* in the popular names table are matchable against our complete enumeration of U.S. laws. We find that out of 7,315 cleanly cited laws in the popular names table, we are able to match 7,229 to our list of U.S. laws (98.8%). Part of the reason we are not able to do even better is that the popular names table does not always disambiguate page citations to the statute at large by referring to chapter or other suitable citation. As a result, if there are multiple laws on a particular page we cannot automatically tell which popular name goes with which law on that page. If we count those popular names matching to more than one law as a “hit” in our query, it turns out we find 7,284 matches, which is to say 99.6% of all the laws we search for can be found in our database.

## Usage Notes

Our database presents a comprehensive table of U.S. laws. Depending on the research task, this table may receive different uses, and it is hard to anticipate all the places it will go. Having said that, we anticipate that scholars will frequently use this dataset by merging in some other dataset with labels, text, and so forth. Their research questions will presumably focus on the analysis of this meta-data about laws, possibly one at a time or perhaps in inter-relationship to one another. The research task about number of laws enacted during “divided government” versus unified government is typical, for example, although perhaps too simple to see what the real challenge is because assigning the “divided government” label requires the use of the “date” column. More challenging and typical would be adding labels that depend on individual identification of laws, for example the “important” versus “unimportant” distinction mentioned earlier. We presume that the scholar interested in our dataset has some set of laws ${\mathscr{X}}$ that they wish to merge to our data in order to compare laws that are in their set to laws that are outside their set. They would do so to illustrate what proportion of all laws their set of particular laws cover, for example, or to examine how some trends differ in their set versus outside their set differ. Often, scholars will have more than one set of laws that they are interested in, and their goal will be to setup a dataset involving sets of laws ${\mathscr{X}}$ and ${\mathscr{Y}}$. When working with multiple sets of partially overlapping lists of laws, it will often make sense to join both against a more comprehensive and universal set, as that keeps the scholars analytical options open. The basic question for using this data is what scholar needs to have in their source data to be able to use it.

On the one hand, we provide a numeric identifier that scholars can include to unambiguously refer to a particular federal law. Moreover, we outline a procedure that should lead to consistent numeric identification even by other scholars on a going forward basis. While providing this identifier is generally a good idea to remove doubt about what law one is referring to, we understand that adoption of our identifiers by other scholars cannot be presumed. Additionally, if procedures were to change or their were additions, amendments, or deletions to future versions of our dataset, then there would be questions about backward compatibility.

Therefore, in addition to referencing the identification system for laws we propose, we would also encourage scholars to pay attention to the citation systems as used in the source documents and record the necessary data accordingly. In particular, we recommend recording data and using matching based on the piece-wise rules as outlined in the methods section above and implemented via special merging functions in the git repository. Beginning with the 86th Congress laws should be matched using the public law number. Prior to the 56th Congress, laws should be matched by chapter number, Congress, and session. Finally, particular care needs to be paid in the matching of laws enacted between the 57th and 85th Congress. Generally, if public laws and chapters will be used to refer to laws in a dataset that is not exclusively relating to recent laws, one *must* also include the Congressional session (or equivalently include the date of enactment, from which one can infer these details). While one often *can* proceed to do matching without all these items, one runs a risk of problems, for example over-matching or mis-matching. More information such as bill title can certainly help to avoid ambiguities, but increasingly one will have a system of citation that is hard to make work across datasets.

Finally, a brief word on enriching these data with full texts of U.S. laws. This dataset does not provide the texts of laws that are listed, although doing so would be an appropriate extension of this work. What this data set does provide is citation information on publicly available source documents, so typically linking the text once found is no more challenging than any other linking of label task described above. That said, getting clean texts of law is very difficult on a consistent basis from public sources. Post-1995 laws in electronic text format are available from the US Government Printing Office (https://www.govinfo.gov/app/collection/plaw). These laws are formatted using markup that allows one to place footnotes correctly and also separate various formatting materials, although at present writing it is not clear how consistently these formats have been used over the years. Since 2013, laws are also available in an XML markup format. Earlier laws in the *Statutes at Large* that are still provided by the US GPO as PDFs, although for most text data projects one would need to then OCR the documents and this generates familiar issues. In terms of comprehensive sources of earlier laws, HeinOnline provides full texts of all laws in both PDF and OCRed text format. Proquest Congressional covers the Statutes at Large between 1789 and 2014, and also offers PDFs (https://proquest.libguides.com/congressionalhelp/ccc). The only comprehensive public source for obtaining law texts of which we are aware is a viewing tool provided by the Office of Revision Counse. To find 14 Stat. 41, for example, one can use this URL and it will generate an appropriate scan (https://uscode.house.gov/statviewer.htm?volume=14 page = 41). Yet, the comprehensiveness of this data is untested at this point and the format is scanned PNGs rather than text or even PDF. Taking it as given that one can find the Statute at Large volume one wants, the citation information provided in the table is sufficient to conduct a table of contents search. Our data also does provide the page and volume number when readily available. Figure 1 shows the table of contents of the statutes at large from both the “early” and “middle” citation regimes^11^.

## Data Availability

All code used to create the dataset from original sources, validate the dataset, as well as generate the figures are available at OSF repository (https://osf.io/mrghc).
